# Letter from the Editor in Chief

**DOI:** 10.19102/icrm.2018.091202

**Published:** 2018-12-15

**Authors:** Moussa Mansour


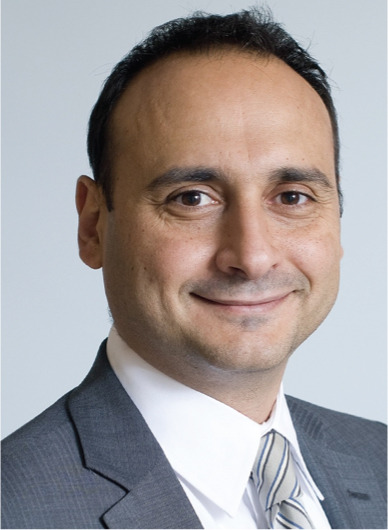


Dear Readers,

This issue of *The Journal of Innovations in Cardiac Rhythm Management* contains an elegant review that I wish to highlight written by Dr. James Reiffel and titled “Oral Anticoagulation and Antiarrhythmic Drug Therapy for Atrial Fibrillation.”^1^ In this article, the author provides a comprehensive summary of the most significant developments in the field of pharmacological treatment of atrial fibrillation (AF), including specifically with respect to oral anticoagulation.

Despite all of the existing developments in the area of anticoagulation for AF and the overwhelming evidence from randomized clinical trials and real-world experience confirming the generalizability of the findings in larger populations, a significant number of patients with AF are still not prescribed anticoagulation. Data from registries such as PINNACLE and others suggest that more than 40% of patients with AF and a CHADS_2_ score of 2 or more are not on anticoagulation. The reason for the underutilization of anticoagulation is likely multifactorial, and improving the situation would probably involve introducing efforts to educate both patients and heath care providers alike. This initiative would likely also include automated best-practice alerts integrated with electronic heath records, allowing for the rapid identification of patients with AF who are at risk of stroke and not on anticoagulation. Such efforts are expected to reduce the incidence of stroke, save lives, and provide significant health care cost benefits and so should be strongly considered for implementation in the future.

On a separate note, I would like to use this opportunity to thank Dr. Van Hare for his service to *The Journal of Innovations in Cardiac Rhythm Management* and welcome his replacement, Dr. Kathryn Collins, who will be taking over as the new Pediatric Electrophysiology section editor for the journal. Dr. Collins has been the Director of Pediatric Electrophysiology at Children’s Hospital Colorado in Aurora, CO since 2007. In practice, she sees a wide variety of patients, from fetal life to adult patients with complex congenital heart disease and arrhythmias. She recently concluded her service on the Pediatric and Congenital Electrophysiology Society Leadership Executive Team. She continues to serve on the Educational Committee of the Heart Rhythm Society. Her research interests include radiofrequency and cryoablation of arrhythmia substrates in pediatric and congenital heart disease. She has published multiple articles on the subject of cryoablation in atrioventricular nodal reentrant tachycardia and also has been a leader in multicenter collaborative research within the PACES organization, primarily with her research on congenital junctional ectopic tachycardia and posterior fascicular ventricular tachycardia in pediatric patients. In the area of cardiac implantable electronic devices, she has been a proponent of alternative implantation techniques for improved cosmetic and quality-of-life outcomes in young patients. Her newest endeavors are in the areas of risk stratification of Wolff–Parkinson–White syndrome in pediatric patients and the evaluation of pediatric patients with midexertional syncope.

With the holiday season upon us, I also want to wish you a happy and healthy new year.

Sincerely,


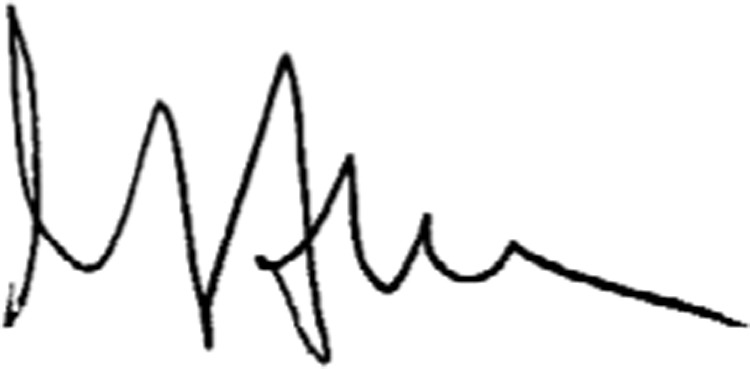


Moussa Mansour, md, fhrs, facc

Editor in Chief

The Journal of Innovations in Cardiac Rhythm Management

MMansour@InnovationsInCRM.com

Director, Atrial Fibrillation Program

Jeremy Ruskin and Dan Starks Endowed Chair in Cardiology

Massachusetts General Hospital

Boston, MA 02114
